# Spatially Resolved Diffusion NMR for Structurally Heterogeneous Materials

**DOI:** 10.1021/acs.analchem.6c00954

**Published:** 2026-07-07

**Authors:** Todor T. Koev, Haider Hussain, Karina Gukhool, Dave J. Adams, Matthew Wallace

**Affiliations:** † School of Chemistry, Pharmacy and Pharmacology, 6106 University of East Anglia, Norwich Research Park, Norwich NR4 7TJ, U.K.; ‡ School of Chemistry, 3526 University of Glasgow, Glasgow, Scotland G12 8QQ, U.K.

## Abstract

Understanding the internal architecture of hydrogel materials is essential for their effective use in biomedical and pharmaceutical applications, yet the use of noninvasive, spatially resolved methods remains limited. We report a robust analytical approach using spatially resolved pulsed-field gradient nuclear magnetic resonance (PFG-NMR) spectroscopy to quantify the depth-dependent self-diffusion of small molecular probes in intact hydrogel systems. By introducing probes post-gelation via passive downward diffusion, this method avoids perturbations associated with probe incorporation during gel formation and enables nondestructive profiling of internal gel architecture. Applied to high amylose maize starch, agarose, and calcium-triggered low-molecular-weight (LMWG) gels, the technique revealed vertical variations in network density and porosity in starch gels, corroborated by scanning electron microscopy, while the other gels exhibited uniform structure. In contrast, conventional nonselective PFG-NMR yields a single self-diffusion coefficient averaged over the entire sample and is unable to reveal the heterogeneity present. Our methodology broadens the NMR analytical toolkit for characterizing soft matter systems and offers promising utility in evaluating structurally complex biomaterials, where spatial heterogeneity is functionally relevant.

## Graphical abstract

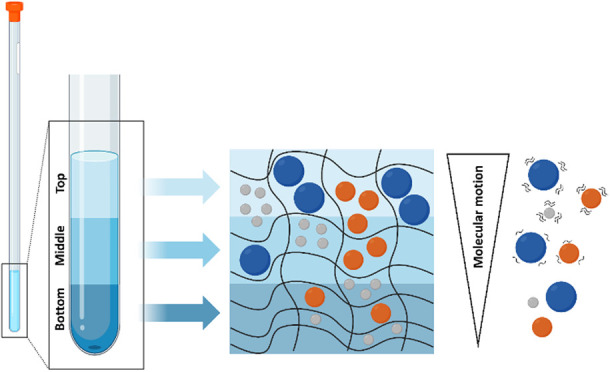

## Introduction

Hydrogels are three-dimensional (3-D) materials, composed of cross-linked hydrophilic polymers forming a large network ensemble, enabling them to hold large amounts of water or biological fluids within their 3-D network. Their hydrophilicity, moldability, swelling, and capillary properties have made them highly promising biomaterials for cosmetic, pharmaceutical, and biomedical applications.^1–3^ The physicochemical and mechanical properties of gel materials, such as solubility, surface charge, tensile strength, and pore size distribution, are important for these materials’ optimal utilization.^4–6^

Preparation methods for the manufacturing of pharmaceutical, biomedical, and cosmetic hydrogels, such as chitosan, alginate, gelatin, cellulose derivatives (*e.g*., hydroxypropyl methylcellulose), and starch, can result in gels featuring inhomogeneous density and pore size distribution, leading to suboptimal and inconsistent material performance. Experimentally, techniques such as scanning and transmission electron microscopy (SEM and TEM, respectively) and atomic force microscopy (AFM) provide detailed information about the heterogeneous internal morphology of such gel systems but are often destructive or deformative in nature. They can also require a lengthy experimental setup and acquisition, as well as extensive sample preparation, which can alter the network morphology of soft gels.^7,8^ Complementary optical approaches such as fluorescence correlation spectroscopy have been used to probe local transport and heterogeneity in hydrogels on the micrometer scale.^9^ However, unless they are repeated throughout a sample, these measurements can fail to capture the macroscopic heterogeneity that can occur in prepared hydrogels.

Previous works have shown that in the absence of specific interactions between small molecular guests and supramolecular networks, the pore size of monodisperse gel networks can be probed indirectly by quantifying the self-diffusion behavior of small molecular probes of different sizes introduced into the network ensemble.^10–15^ However, these methods provide an average pore size estimation, resulting in misleading information when probing heterogeneous gel networks. In this work, we develop a rapid, noninvasive, and nondestructive quantitative toolkit for spatially resolved detection of structural inhomogeneities in gel networks, which appear homogeneous to the naked eye (see photographs, Figure 1). We gain spatially encoded information^16^ on changes in the self-diffusion coefficients of a library of small molecular probes of various hydrodynamic radii (Figure 1b) at different depths of the gel networks, thus rapidly probing vertical inhomogeneities in their internal organization. Spatial resolution is afforded along the vertical axis of the NMR tube, providing a direct readout of depth-dependent structural gradients and probe diffusion while retaining the noninvasive and quantitative advantages of diffusion NMR.

**Figure 1:**
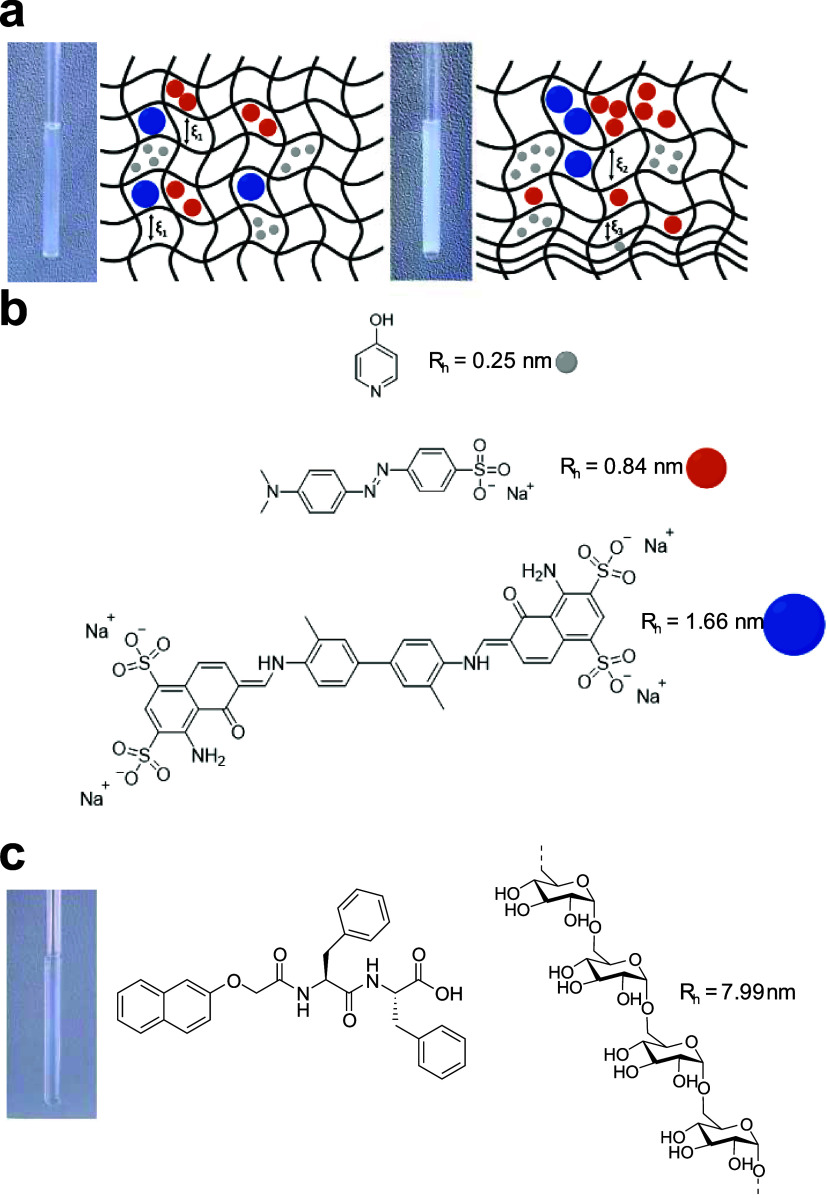
(a) Photos of agarose and maize starch gels, and respective sketches of gel networks with homogeneous (ξ_1_ = ξ_1_, left) and inhomogeneous (ξ_2_ > ξ_3_, right) pore size distributions along their length, with small molecular probes of varying hydrodynamic radii (b) diffusing in a less restricted (orange) and more restricted (blue) manner within the gel networks. Molecular probes 4-hydroxypyridine, methyl orange, and Evans blue, and their hydrodynamic radii (*R*_h_, b). Photo of gel formed from naphthalene-functionalized dipeptide, 2NapFF, in an NMR tube, structures of dipeptide and dextran (c).

We apply these methods to high amylose maize starch hydrogels (AM) which are prone to sedimentation and inhomogeneous pore size distribution, and agarose gels which have homogeneous pore sizes.^17,18^ We also study dipeptide low-molecular-weight gelators (LMWGs),^19^ triggered by the downward diffusion of CaCl_2_ into a sample, and investigate the combination of spatially resolved PFG-NMR with ^2^H NMR to study vertical homogeneity. These peptide-based materials represent an important class of advanced materials for the delivery of proteins, where structural heterogeneities can be expected to have an important influence on release kinetics.^20^ On all samples, we compare our spatially resolved methodology with data obtained from conventional (nonspatially resolved) pulsed-field gradient (PFG) NMR experiments. Hydrogels for analysis by NMR are typically prepared directly in 5 mm sample tubes to preserve integrity and maintain suitable magnetic field homogeneity.^21^ Our findings highlight that appreciable vertical density gradients can develop under these conditions that should be considered when interpreting measurements performed without spatial resolution. Another key advantage of spatial selection is that it enables the probes to be introduced after gel assembly via downward diffusion, where a homogeneous concentration of probe molecules across the sample could not be guaranteed (as required by conventional PFG-NMR experiments). This ability is particularly advantageous for any dynamic supramolecular system where *in situ* probe delivery may alter the scaffolds’ biorelevant properties, or where the incorporation of small molecular probes during gelation may disrupt network formation.^22,23^

## Materials and Methods

### Materials

High amylose maize starch (CAS 9005–82–7), agarose (CAS 9012–36–6), deuterium oxide (D_2_O) (CAS 7789–20–0), 4-hydroxypyridine (CAS 626–64–2), methyl orange (CAS 547–58–0), Evans blue (CAS 314–13–6), CaCl_2_ (CAS 10043–52–4), and dextran (100 kDa, CAS 9004–54–0) were all purchased from Merck (formerly Sigma-Aldrich, Darmstadt, Germany). 2NapFF was synthesized as described elsewhere.^19^

### Gel Preparation

Starch-D_2_O suspensions (10% w/v) were prepared directly in NMR tubes (Wilmad 528-PP), where each starch suspension contained one of three small molecules (4-hydroxypyridine, PYR; methyl orange, MOR; and Evans blue, EVB) at 2.0 mM. Each sample was vortexed and placed on an end-overend mixer (40 rpm) overnight, before being autoclaved (121 °C, 15 psi, 20 min). All gel samples were left to set at 4 °C for 72 h before further analysis.

To prepare agarose gels, agarose-D_2_O suspensions (5% w/v) were prepared directly in NMR tubes containing one of the three guest molecules (2.0 mM). The NMR tubes containing each suspension were placed in a water bath set at 100 °C for 7.5 min, each sample vortexed in the NMR tube (vortex genie 2, 3200 rpm, 10 s) and returned to the water bath (100 °C for 7.5 min). The resulting solutions were placed at 4 °C for 72 h before further handling.

To investigate the addition of probe molecules after gelation, starch gels were prepared analogously to above in NMR tubes without any small molecular probes. Following gel setting (4 °C, 72 h), 100 μL of EVB (12 mM in D_2_O) was pipetted on top of the set gels and allowed 48 h to diffuse to the bottom of each gel. To prepare starch gels for microscopy, 10% starch-water suspensions were placed in screw-cap glass vials, placed on an end-overend mixer overnight, and autoclaved. Starch gels were left to set (4 °C, 72 h).

To prepare gels of the dipeptide, 2NapFF, 6 mg/mL solutions were prepared in 10 vol % D_2_O/90% H_2_O by stirring solid dipeptide for 24 h with NaOH (0.014 M). NMR samples were prepared by combining the 2NapFF solution with either H_2_O/D_2_O or dextran solution (100 kDa) in a 5 mm NMR tube (Wilmad 528-PP-7) to achieve concentrations of 5 and 10.8 mg/mL of 2NapFF and dextran, respectively. CaCl_2_ (40 μL, 0.75 M) was then added on top using an extended-length polypropylene pipet tip (Starlab), and the sample was immediately loaded into the NMR spectrometer (500 MHz). After 24 h, the samples were removed from the spectrometer and placed in a water bath at 25 °C. After 2 weeks, dextran solution (60 μL, 100 kDa, 100 mg/mL) was placed on top of the sample, which did not have dextran added previously, and stood for 1 month at 25 °C before analysis.

### Microscopy

Gels were rinsed with cold distilled deionized water (*dd*H_2_O), laterally placed in 2.5 mL embedding plastic boats, and covered in mounting medium (PolyFreeze O.C.T. medium, Merck SHH0026). The mounting medium was fully solidified in an EtOH/dry ice bath; the samples were wrapped in aluminum foil and placed at −80 °C until further handling.

Cryoset samples were mounted on cryostubs and sectioned on a CryoStat (Thermo Cryostar NX70), equilibrated at −10 °C, at 10 μm thickness, and placed directly on scanning electron microscopy stubs, covered in double-sided adhesive carbon conducting tape. The samples were coated in gold and visualized by a Zeiss EVO scanning electron microscope at a 3 kV electron high tension and imaged at 1000- and 2500-fold magnification.

### NMR Experiments

All experiments were performed at 298 K on a Bruker Avance Neo spectrometer, operating at 499.31 MHz ^1^H frequency, equipped with a Bruker iProbe TBO, with a maximum pulsed-field gradient strength, *g*_max_, of 50 G cm^–1^, confirmed using a standard sample of 1% H_2_O in D_2_O.

### Diffusion-Ordered Spectroscopy (DOSY)

Conventional PFG-NMR experiments to study probe diffusion were performed using a stimulated echo sequence with bipolar gradient pulses (Bruker library, *stebpgp1s*). In all experiments, the probes’ peak intensity, I, was recorded as a function of 16 different gradient strength (*g*) values (2 to 98% *g*_max_), obtaining a minimum of >92% attenuation of the probes’ signals. All experiments were performed using a minimum of 16 scans per gradient strength value, an acquisition time of 1.4 s, a relaxation delay of 6 s, a diffusion duration, Δ, of 0.25 s, a gradient pulse in the shape of a smoothed square with a combined length, δ, of 1.5 ms for the bipolar pair, and a gradient recovery delay, τ, of 200 μs.

Self-diffusion coefficients were taken as the arithmetic mean obtained by integration of the separate resonances of each probe as a function of gradient strength (7.81, 6.52 ppm PYR; 7.82, 7.61, 6.65 ppm MOR; 8.25, 7.73, 7.55 ppm EVB) and fitting to eq 1:

1 $$I=I_{0}\;\exp\left(-D\gamma^{2}g^{2}\delta^{2}\left(\Delta -\frac{\delta }{3}-\frac{\tau }{2}\right)\right)$$

### Spatially Resolved DOSY (SR-DOSY)

Spatially resolved excitation pulsed-field gradient NMR^16,24^ (†ESI) experiments were performed on all small-molecule-loaded gels. The depth along the z-line (the NMR tube) was varied using the frequency offset of the slice-selective refocusing shaped pulse (*spoffs1*), with a spatially resolved window width of *ca*. 4 cm (*spoffs1* −20 to 20 kHz). The z-gradient power (*gpz1*) was 5% (2.5 G cm^–1^), using π/2 *rf* of 8.0 μs, recycle delay of 2 s, gradient pulse of 6000 μs, and a diffusion delay of 25 ms. A 2 s presaturation pulse (41.78 dB) was applied for water suppression. Self-diffusion coefficients of our probes are thus determined over a slice width of approximately 1 mm, observing the aromatic resonances of the probes listed above.^24^ Spatially selective self-diffusion coefficients were averaged across the top (25–35 mm), middle (15–25 mm), and bottom (5–15 mm from the absolute bottom of the NMR tube) to obtain NMR tube region average self-diffusion coefficients, fitting the data to eq 2.

2 $$I=I_{0}\;\exp\left(-D\gamma^{2}g^{2}\delta^{2}\left(\Delta -\frac{\delta }{3}\right)\right)$$

### Spatially Resolved ^2^H Chemical Shift Imaging

^2^H chemical shift imaging experiments were performed using the gradient phase encoding sequence of Trigo-Mouriño et al.^25^ in 32 slices with 1 scan and acquisition and relaxation times of 2.6 and 1 s, respectively.

### Pore Size Estimation

The pore size in both starch and agarose gels was estimated from the restriction of the diffusivity of probes within the gel network (eq 3).

3 $$\frac{D}{D_{0}}=\exp\left[-\frac{R_{h}}{\xi }\right]$$

where *D*/*D*_0_ is the restriction in diffusivity expressed as the quotient ratio of the probe’s diffusivity within the gel network and its diffusivity in the absence of a gel network; *R*_h_ is the probe’s hydrodynamic radius; and ξ is a representative pore size for the material.^26^

## Results and Discussion

Internal organization and porosity of macromolecular systems have been shown to be important factors for their directed applications.^27^ On comparison of the self-diffusion coefficients of PYR, MOR, and EVB in a 10% starch gel, it was observed that all three guest molecules exhibited different self-diffusion coefficients at the top (25–35 mm from the base of the NMR tube), middle (15–25 mm), and bottom (5–15 mm) of the starch gel system. All three guest molecules exhibited the least restricted behavior at the top and the most restricted at the bottom of the gel (Figures 2a,b, S1, and S2, Supporting Information).

**Figure 2:**
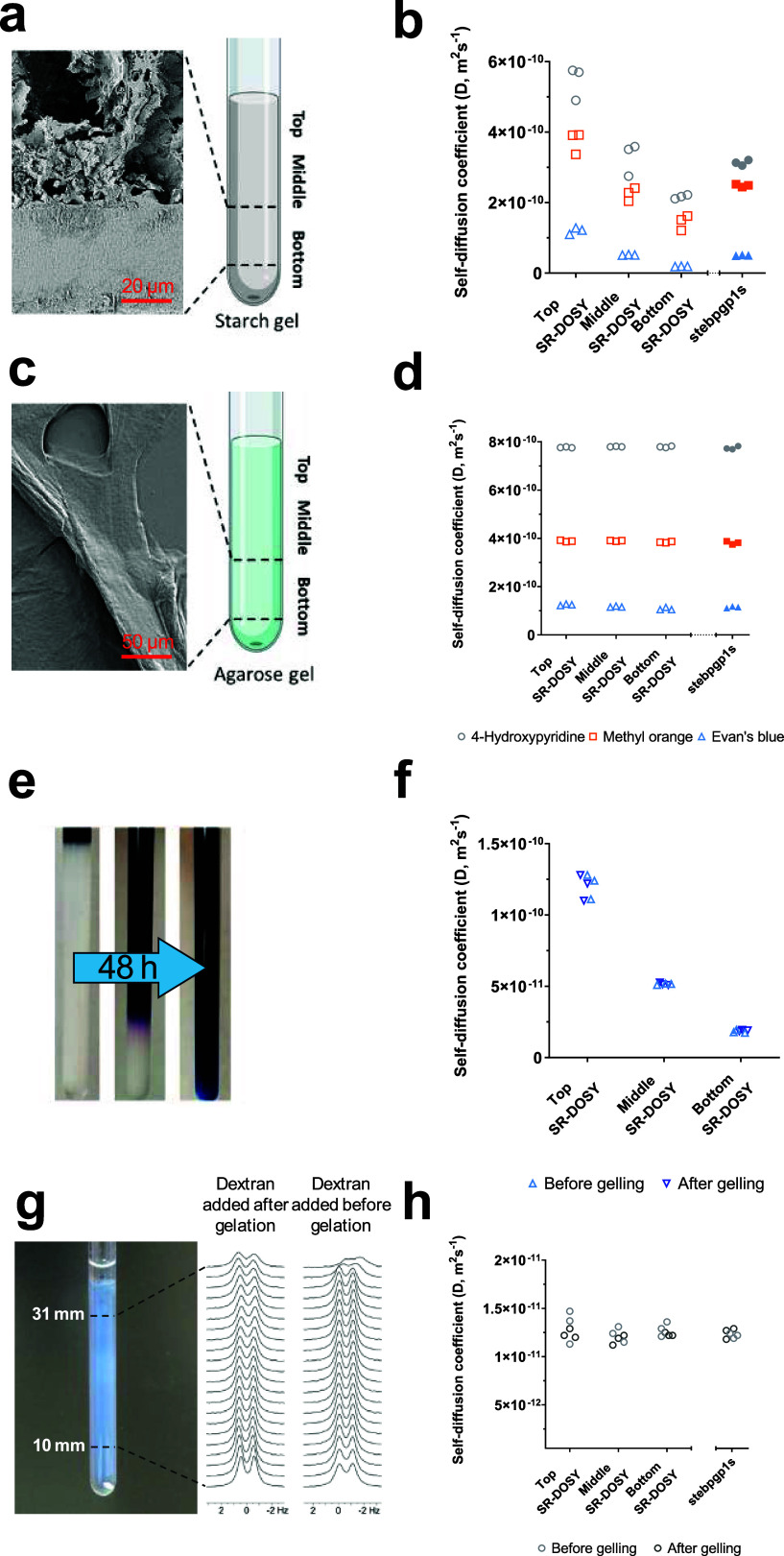
Scanning electron microscopy images of 10% starch and 5% agarose gels (a, c). Self-diffusion coefficients of 4-hydroxypyridine (gray), methyl orange (orange), and Evans blue (blue) using spatially resolved (SR) and nonselective PFG-NMR (*stebpgp1s*) in starch (b) and agarose gels (d), *n* = 3. Comparison of self-diffusion coefficients of Evans blue introduced before and after gelling, measured 48 h after probe introduction (e) of a 10% starch gel, using SR PFG-NMR (f). Photos of low-molecular-weight self-assembling gel (g) and inlay showing spatially resolved ^2^H NMR spectra of dipeptide gels with dextran added after (left) and before (right) gelation. Self-diffusion coefficients of dextran (100 kDa) using SR and nonselective PFG-NMR (h), *n* = 3.

This observation was interpreted as the starch gel system undergoing sedimentation effects following its gelatinization stage, which resulted in a higher network density and compaction near the bottom of the gel. This was further evidenced by SEM images recorded along the length of the starch gels showing a higher density near the bottom of the hydrogel (Figure 2a, left). In contrast, a 5% agarose gel did not exhibit significant differences in the self-diffusion coefficients of the three guest molecules between the top, middle, and bottom of the sample (Figure 2c,d).

Using EVB as a reference molecular probe and fitting to eq 3 (Figure S3, Supporting Information), the pore size distribution of starch and agarose gels was found to be in the ranges of 0.9–40 and 24–37 nm, respectively (Figure 3), highlighting the much greater heterogeneity in internal organization of starch vs agarose gels. A pore size of 30 nm is in close agreement with values of ca. 50 nm reported by Narayanan et al. in 3 wt % agarose gels.^17^ The pore size of the dipeptide gels was found to be in the range of 40–57 nm, in agreement with previous measurements on this system.^11^

**Figure 3:**
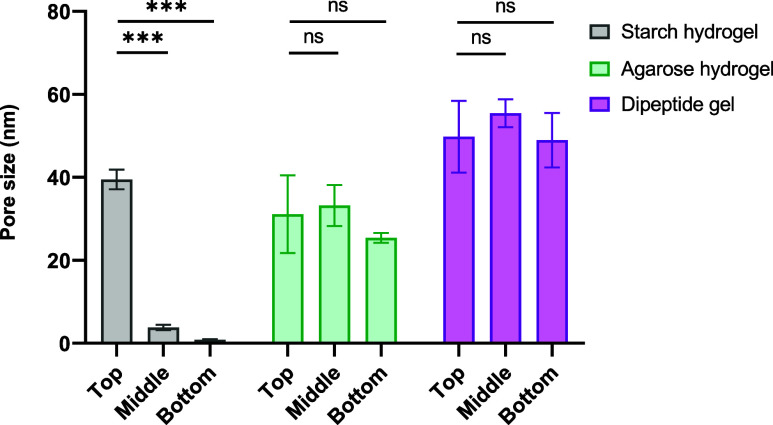
Average pore size measurements for starch, agarose, and dipeptide gels along three different depth regions of the gels using EVB and dextran as a diffusivity probe, *n* = 3, *** *p* < 0.001, ** *p* < 0.01.

Previous works have demonstrated the use of nonselective PFG-NMR to measure diffusion of guest molecules in a large network ensemble, and extract information on the macromolecular system’s porosity.^10^ On comparison of the self-diffusion coefficients of PYR, MOR, and EVB in the inhomogeneous (i.e., starch) gels, it is apparent that self-diffusion coefficients obtained using conventional PFG-NMR are in-between those recorded using spatially resolved experiments (Figure 2b). We note that in analogy to adenosine triphosphate (ATP), the diffusion of these anionic probes (MOR, EVB) is expected to depend on obstruction by the gel network rather than specific charge-dependent adsorption.^28^ In homogeneous gels (agarose), both spatially resolved and nonselective experiments yielded self-diffusion coefficients in close agreement with each other (Figure 2d). Similarly, dipeptide gels prepared in the magnetic field (11.4 T) of the NMR spectrometer exhibit a uniform ^2^H residual quadrupolar coupling (*ca*. 1.1 Hz) along their length, indicating a uniform network structure and degree of alignment of the gel fibers that is independent of whether the dextran probe was added before or after gelation (Figure 2g).^29^ These results validate the use of Ca^2+^ to induce the homogeneous gelation of these dipeptide materials and investigate their porous architecture via the diffusion of imbibed polymer probes. Together our data highlights the utility of spatially resolved techniques when probing soft matter systems’ internal organization where sedimentation or phase separation may occur, and conventional NMR would provide only an average and potentially misleading analysis of the network organization across the entire material.

Understanding structural inhomogeneities, such as differences in macromolecular architecture, can be highly advantageous for the better understanding of 3-D cell culture scaffolds and biofilm treatment strategies.^30,31^ However, introducing small molecular probes during the formation of such systems is not viable, due to the possible impact on macromolecular structure and organization.^32^ The same is true of many synthetic hydrogel systems and self-assembling materials where including the probes during gelation may interfere with the self-assembly process. Nevertheless, the self-diffusion coefficients of EVB in a 10% starch gel and 100 kDa dextran in a dipeptide gel were comparable irrespective of whether the probe was introduced before or after gelation (Figure 2f,h), allowing us to verify that the presence of the molecular probes does not interfere with network formation. Furthermore, this comparison highlights the method’s suitability to preformed network ensembles and biologically relevant systems.

## Conclusions

This work demonstrates that spatially resolved PFG-NMR provides a robust, noninvasive means of probing internal heterogeneity in soft matter systems that appear macroscopically homogeneous. By resolving depth-dependent self-diffusion, the method reveals sedimentation- and compaction-driven structural gradients in starch gels that are obscured by conventional, spatially averaged PFG-NMR, while correctly identifying uniform architectures in agarose and calcium-triggered dipeptide gels. A significant advantage of the approach is the ability to introduce probe molecules after gel formation, preserving native network structure and enabling diffusion measurements in systems where probe incorporation during gelation is undesirable or impractical. This nondestructive approach should be particularly valuable for gels that undergo kinetic or thermodynamic evolution over time (*e.g*., syneresis in starch gels) with changes in organization along the vertical axis. By remeasuring probe self-diffusion in the same sample over several days, spatially resolved PFG-NMR could enable time-dependent changes in gel architecture to be followed directly. This capability broadens the applicability of diffusion NMR to biologically and pharmaceutically relevant hydrogels including self-assembling and responsive materials. In the absence of triple-axis pulsed-field gradients, the method is sensitive only to the porosity variation along the vertical axis of the NMR tube. For samples prepared directly in the tube, lateral heterogeneity may arise from wall effects or syneresis, and researchers should remain alert to these possibilities. Complementary techniques, such as dye staining and light microscopy, are recommended to verify lateral homogeneity prior to NMR measurements. Additionally, care should be taken to account for the size and center position of the NMR detection window, which are defined by the length of the RF coil in the spectrometer. Validation using biphasic samples is advised to confirm appropriate window calibration.^33^ Overall, spatially resolved diffusion NMR expands the analytical toolkit for soft matter characterization by directly linking local transport behavior to internal structure. As functionally graded and heterogeneous hydrogels become increasingly important, this methodology offers a valuable route to nondestructive structural assessment and materials optimization.

## Supplementary Material



## Notes

Pulse code for spatially resolved DOSY NMR experiment (PDF)

## References

[1] Rajwade J. M., Paknikar K. M., Kumbhar J. V. (2015). Applications of Bacterial Cellulose
and Its Composites in Biomedicine. Appl. Microbiol.
Biotechnol..

[2] Torres F. G., Commeaux S., Troncoso O. P. (2012). Biocompatibility
of Bacterial Cellulose
Based Biomaterials. J. Funct. Biomater..

[3] Koetting M.
C., Peters J. T., Steichen S. D., Peppas N. A. (2015). Stimulus-Responsive
Hydrogels: Theory, Modern Advances, and Applications. Mater. Sci. Eng. R Reports.

[4] De
France K. J., Xu F., Hoare T. (2017). Structured Macroporous
Hydrogels - Progress, Challenges, and Opportunities. Adv. Healthcare Mater..

[5] Lee S. H., Shim K. Y., Kim B., Sung J. H. (2017). Hydrogel-based Three-dimensional
Cell Culture for Organ-on-a-chip Applications. Biotechnol. Prog..

[6] Annabi N., Nichol J. W., Zhong X., Ji C., Koshy S., Khademhosseini A., Dehghani F. (2010). Controlling the Porosity and Microarchitecture
of Hydrogels for Tissue Engineering. Tissue
Eng. Part B: Rev..

[7] Schillers H., Rianna C., Schäpe J., Luque T., Doschke H., Wälte M., Uriarte J. J., Campillo N., Michanetzis G. P. A., Bobrowska J., Dumitru A., Herruzo E. T., Bovio S., Parot P., Galluzzi M., Podestà A., Puricelli L., Scheuring S., Missirlis Y., Garcia R., Odorico M., Teulon J.-M., Lafont F., Lekka M., Rico F., Rigato A., Pellequer J.-L., Oberleithner H., Navajas D., Radmacher M. (2017). Standardized
Nanomechanical Atomic Force Microscopy Procedure (SNAP) for Measuring
Soft and Biological Samples. Sci. Rep..

[8] Vieira V. M. P., Hay L. L., Smith D. K. (2017). Multi-Component
Hybrid Hydrogels
– Understanding the Extent of Orthogonal Assembly and Its Impact
on Controlled Release. Chem. Sci..

[9] Lehmann S., Seiffert S., Richtering W. (2012). Spatially
Resolved Tracer Diffusion
in Complex Responsive Hydrogels. J. Am. Chem.
Soc..

[10] Leloup V. M., Colonna P., Ring S. G. (1990). Studies on Probe Diffusion and Accessibility
in Amylose Gels. Macromolecules.

[11] Wallace M., Adams D. J., Iggo J. A. (2013). Analysis
of the Mesh Size in a Supramolecular
Hydrogel by PFG-NMR Spectroscopy. Soft Matter.

[12] Löser L., Bunk C., Scholz R., Lang M., Böhme F., Saalwächter K. (2024). Structural Characterization of Amphiphilic Conetworks
in Selective and Nonselective Solvents Using 1H NMR and SAXS. Macromolecules.

[13] Jowkarderis L., Ven T. G. M. (2015). Mesh Size Analysis
of Cellulose Nanofibril Hydrogels
Using Solute Exclusion and PFG-NMR Spectroscopy. Soft Matter.

[14] Le
Feunteun S., Mariette F. (2007). Impact of Casein Gel Microstructure
on Self-Diffusion Coefficient of Molecular Probes Measured by 1H PFG-NMR. J. Agric. Food Chem..

[15] Morbidini R., Edkins R. M., Carrascosa-Tejedor J., Czakkel O., Hanafy B. I., Kalaria D. R., Seydel T., Edkins K. (2026). Comparing Microscopic
and Macroscopic Diffusion in Drug Delivery: A Study of Small Drug
and Protein Dynamics in a Supramolecular Peptide Hydrogel. J. Colloid Interface Sci..

[16] Wisniewska M. A., Seland J. G. (2019). Investigating Structure-Dependent
Diffusion in Hydrogels
Using Spatially Resolved NMR Spectroscopy. J.
Colloid Interface Sci..

[17] Narayanan J., Xiong J.-Y., Liu X.-Y. (2006). Determination of
Agarose Gel Pore
Size: Absorbance Measurements Vis a Vis Other Techniques. J. Phys.:Conf. Ser..

[18] Koev T. T., Muñoz-García J. C., Iuga D., Khimyak Y. Z., Warren F. J. (2020). Structural Heterogeneities
in Starch Hydrogels. Carbohyd Polym..

[19] Wallace C. M., Rovers M. M., Bellan R., Rutten M. G. T. A., Seddon A., Dalby M. J., Dankers P. Y. W., Adams D. J. (2024). Investigating
the Self-Assembly of 2NapFF and Ureido-Pyrimidinone Multicomponent
Systems for Cell Culture. J. Mater. Chem. B.

[20] Bianco S., Hasan M., Ahmad A., Richards S.-J., Dietrich B., Wallace M., Tang Q., Smith A. J., Gibson M. I., Adams D. J. (2024). Mechanical Release
of Homogenous Proteins from Supramolecular
Gels. Nature.

[21] Tolkkinen K., Mankinen O., Mailhiot S. E., Telkki V.-V. (2024). Ultrafast T 1–T
1ρ NMR for Correlating Different Motional Regimes of Molecules. Anal. Chem..

[22] Alexander S. L. M., Korley L. T. J. (2018). Nucleation Effects
of High Molecular Weight Polymer
Additives on Low Molecular Weight Gels. Polym.
J..

[23] Pont G., Chen L., Spiller D. G., Adams D. J. (2012). The Effect
of Polymer
Additives on the Rheological Properties of Dipeptide Hydrogelators. Soft Matter.

[24] Mitrev Y., Simova S., Jeannerat D. (2016). NMR Analysis
of Weak Molecular Interactions
Using Slice-Selective Experiments via Study of Concentration Gradients
in Agar Gels. Chem. Commun..

[25] Trigo-Mouriño P., Merle C., Koos M. R. M., Luy B., Gil R. R. (2013). Probing
Spatial Distribution of Alignment by Deuterium NMR Imaging. Chem. - Eur. J..

[26] Langevin D., Rondelez F. (1978). Sedimentation of Large Colloidal Particles through
Semidilute Polymer Solutions. Polymer.

[27] Hernandez J. L., Woodrow K. A. (2022). Medical Applications of Porous Biomaterials: Features
of Porosity and Tissue-Specific Implications for Biocompatibility. Adv. Healthcare Mater..

[28] Majer G., Southan A. (2017). Adenosine Triphosphate
Diffusion through Poly­(Ethylene
Glycol) Diacrylate Hydrogels Can Be Tuned by Cross-Link Density as
Measured by PFG-NMR. J. Chem. Phys..

[29] Wallace M., Cardoso A. Z., Frith W. J., Iggo J. A., Adams D. J. (2014). Magnetically
Aligned Supramolecular Hydrogels. Chem. - Eur.
J..

[30] Wimpenny J., Manz W., Szewzyk U. (2000). Heterogeneity
in Biofilms. FEMS Microbiol. Rev..

[31] Xie J., Bao M., Bruekers S. M. C., Huck W. T. S. (2017). Collagen Gels
with Different Fibrillar Microarchitectures Elicit Different Cellular
Responses. ACS Appl. Mater. Interfaces.

[32] Zhou C., Zhou Y., Zheng Y., Yu Y., Yang K., Chen Z., Chen X., Wen K., Chen Y., Bai S., Song J., Wu T., Lei E., Wan M., Cai Q., Ma L., Wong W.-L., Bai Y., Zhang C., Feng X. (2023). Amphiphilic Nano-Swords for Direct Penetration and Eradication of
Pathogenic Bacterial Biofilms. ACS Appl. Mater.
Interfaces.

[33] Wallace M., Adams D. J., Iggo J. A. (2018). Titrations without the Additions:
The Efficient Determination of pK a Values Using NMR Imaging Techniques. Anal. Chem..

