# Supplementary material for: Spatially Resolved Diffusion NMR for Structurally Heterogeneous Materials
Source: Anal Chem. 2026 Jul 7;98(28):20761–7. doi: 10.1021/acs.analchem.6c00954 (PMC13393084; doi:10.1021/acs.analchem.6c00954)
Supplement: Supplementary file 1 [file ac6c00954_si_001.pdf]

# Supporting information for: Spatially resolved diffusion NMR for structurally heterogeneous materials

Todor T. Koev*[1], Haider Hussain[1], Karina Gukhool[1], Dave J. Adams[2] and Matthew Wallace*[1]

1. School of Chemistry, Pharmacy and Pharmacology, University of East Anglia, Norwich Research Park, NR4 7TJ, UK
2. School of Chemistry, University of Glasgow, Glasgow, G12 8QQ, Scotland, UK

**t.koev@uea.ac.uk**

matthew.wallace@uea.ac.uk

# Contents

S1. Spatially selective DOSY pulse sequence ....................................................2

S2. Self-diffusion coefficients of small molecular probes per millimetre of gel depth...............................................................................................................4

S3. PFG-NMR signal decay curves for EVB in free solution and starch gels. .....5

### S1. Spatially selective DOSY pulse sequence

```
;Slice selective refocusing pulse in presence of gradient for spatially resolved DOSY 2D
;MW UEA, February 2021
;Edited by TK and HH, September 2022
;avance-version (12/01/11)

;$CLASS=HighRes
;$DIM=1D
;$TYPE=
;$SUBTYPE=
;$COMMENT=


#include <Avance.incl>
#include <Grad.incl>
#include <Delay.incl>

define list<gradient> diff=<Difframp>

"acqt0=0"
"d12=20u"
"DELTA1=d20*0.5-p30*0.5-p12*0.5-400u"
"DELTA2=DELTA1+150u"

1 ze
2 30m
50u BLKGRAMP
d12 pl9:f1
  d1 cw:f1 ph29
  4u do:f1
 d12 pl1:f1
  50u UNBLKGRAMP
  p1 ph1
50u
p30:gp6*diff
DELTA1
200u pl0:f1
;400u either side of shaped pulse
 200u gron2
(p12:sp1 ph2):f1
200u
50u groff
DELTA2
p30:gp6*diff
130u
d12 pl1:f1
  50u
  go=2 ph31
  30m mc #0 to 2 F1QF(calgrad(diff))
 50u BLKGRAMP
```

exit

ph1=0 0 3 3 2 2 1 1
ph2=1 3
ph31=0 0 1 1 2 2 3 3
ph29=0

;pl0 : 0W
;pl1 : f1 channel - power level for pulse (default)
;p1 : f1 channel -  90 degree high power pulse
;p12: f1 channel - Shaped refocussing pulse [4 ms, Gaus1.1000]
;p30: homospoil/gradient pulse
;d1 : relaxation delay; 1-5 * T1
;d12 : delay for power switching
;d16 : delay for homospoil/gradient recovery
;ns : 2 * n, total number of scans: NS * TD0
;p3 : water suppression pulse
;spoffs1 : offset of refocusing pulse, spoffs1 = 0 is centre of sample, then +/-1000 Hz/mm (at gpz1 = 5%, see below) is up or down from centre

;for z-only gradients:
;gpz1: 5%
;gpz2: 5-20%
;at gpz1 = 5%, then spoffs1 ca. 1000 Hz/mm
;gpz6: 100%, DOSY PFG

;use gradient files:
;gpnam6: SMSQ10.100

;$Id: zggpse,v 1.12 2012/01/31 17:49:32 ber Exp $

**S2. Self-diffusion coefficients of small molecular probes per millimetre of gel depth.**

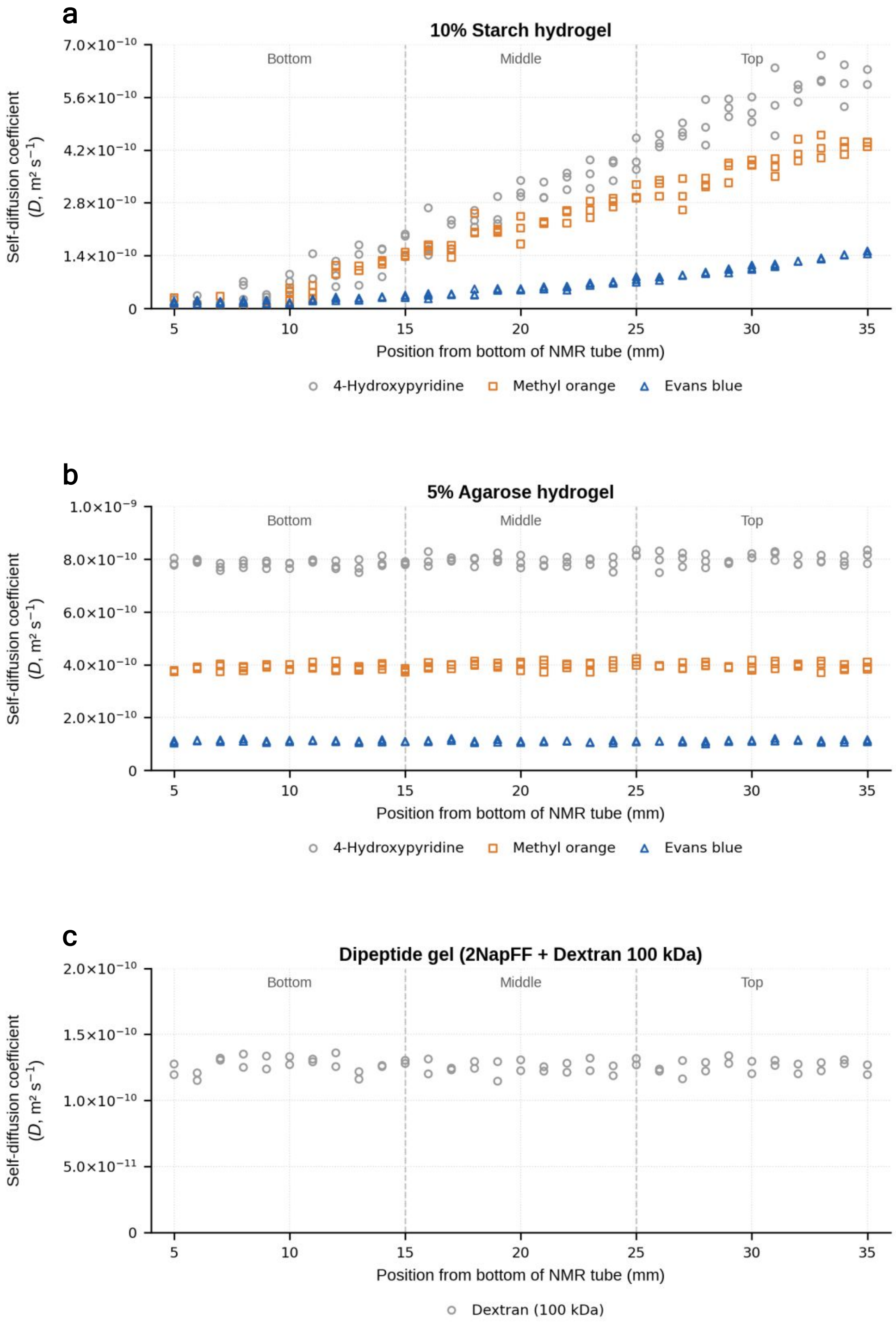


**Figure S1.** Depth-resolved self-diffusion coefficients of molecular probes across starch (a), agarose (b) and dipeptide gels (c) at 1 mm spatial resolution by SR-DOSY NMR.

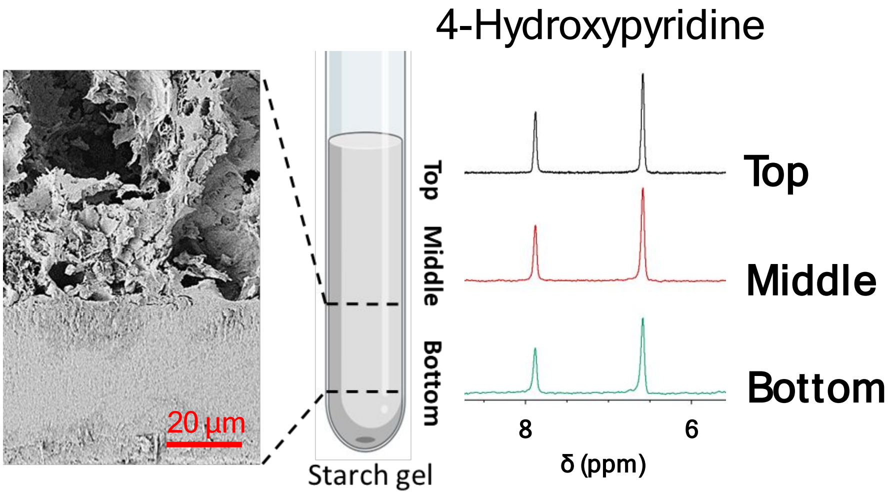


**Figure S2.** $^{1}H$ NMR spectra of the top, middle and bottom layer of the starch gel, showing the aromatic peaks of 4-hydroxypyridine.

### S3. PFG-NMR signal decay curves for EVB in free solution and starch gels.

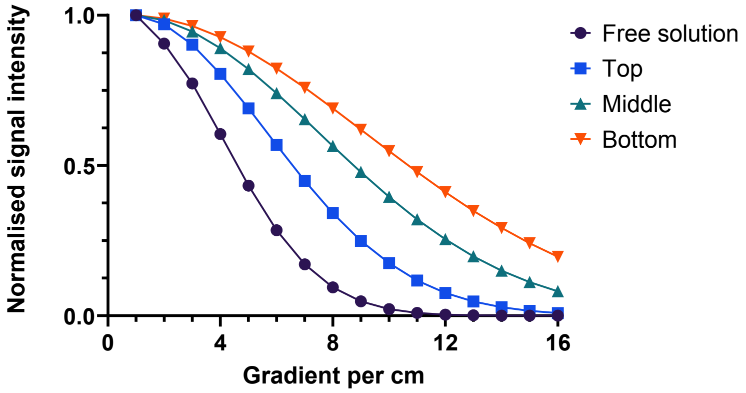


**Figure S3.** Fitting curves for the self-diffusion coefficient for EVB in free solution, top, middle and bottom of the starch hydrogel.
